# Supplementary material for: Spectral consistency in sound sequence affects perceptual accuracy in discriminating subdivided rhythmic patterns
Source: PLoS One. 2024 May 28;19(5):e0303347. doi: 10.1371/journal.pone.0303347 (PMC11132482; doi:10.1371/journal.pone.0303347)
Supplement: S1 Table — (PDF) [file pone.0303347.s002.pdf]

S1 Table. Summary of the linear-mixed model fitted to σ.

Experiment	Variable	*slope*	*SE*	*df*	*t*-value	*p*-value
Exp. 1	frequency	0.26	0.06	38.0	4.33	$p < 0.001$
	order	0.11	0.08	68.1	1.55	$p = 0.126$
	frequency×order	0.02	0.09	38.0	0.19	$p = 0.847$
Exp. 2	frequency	0.14	0.05	41.0	2.51	$p = 0.016$
	order	0.08	0.07	71.6	1.10	$p = 0.277$
	frequency×order	0.12	0.08	41.0	1.49	$p = 0.144$
Exp. 3	frequency	0.34	0.07	28.0	4.77	$p < 0.001$
	order	−0.06	0.09	50.9	−0.59	$p = 0.558$
	frequency×order	0.11	0.11	28.0	0.97	$p = 0.341$
